# The learning community faculty experience: how longitudinal relationships with learners enhance work meaning

**DOI:** 10.1007/s40037-020-00614-z

**Published:** 2020-08-20

**Authors:** Danielle Roussel, Paul R. Gordon, James M. Wagner, Michelle Bardack, Maya G. Sardesai, Jorie M. Colbert-Getz

**Affiliations:** 1 Department of Anesthesiology, University of Utah School of Medicine, Salt Lake City, UT USA; 2 Department of Family & Community Medicine, University of Arizona College of Medicine, Tucson, AZ USA; 3 Department of Internal Medicine, University of Texas Southwestern Medical Center, Dallas, TX USA; 4 Department of Family and Community Medicine, University of New Mexico School of Medicine, Albuquerque, NM USA; 5 Department of Otolaryngology—Head & Neck Surgery, University of Washington School of Medicine, Seattle, WA USA; 6 Department of Internal Medicine, University of Utah School of Medicine, Salt Lake City, UT USA

**Keywords:** Learning communities, Clinical education, Work meaning, Job engagement

## Abstract

**Introduction:**

Work meaning has gained attention as an important contributor to physician job engagement and well-being but little is known about how faculty participation in medical school learning communities might influence this phenomena. Our study goals were to determine how physician faculty members may derive meaning from serving as mentors for longitudinal learning communities of medical students, to understand how that meaning may impact other areas of their work, and relate our findings to existing literature and theoretical frameworks.

**Methods:**

The research team conducted, recorded, transcribed, and coded 25 semi-structured telephone interviews of faculty mentors from four US medical schools with curricular learning communities. The team used an iterative interview coding process to generate final themes and relate these themes to existing literature.

**Results:**

The authors identified five themes of meaning faculty derive from participation as learning community mentors: “I am a better professional,” “I am more connected,” “I am rejuvenated,” “I am contributing,” and “I am honored.” A sixth theme, “I am harmed,” encompassed the negative aspects of the learning community faculty experience. The authors found that their identified themes related closely to the theoretical framework for pathways to meaningful work proposed by Rosso et al.

**Discussion:**

The alignment of the themes we identified on the experience of learning community faculty to existing literature on work meaning corroborates the theoretical framework and deepens understanding of beneficial and harmful learning community effects on faculty. As learning communities become increasingly common within medical schools, this understanding may be important for leaders in academic medicine considering potential indirect benefits of this educational model.

**Electronic supplementary material:**

The online version of this article (10.1007/s40037-020-00614-z) contains supplementary material, which is available to authorized users.

## Introduction

Work meaning has been recognized as an important contributor to job engagement which organizational psychology literature suggests is the positive antithesis to burnout [1, 2]. Physician burnout has received considerable attention due to its association with increased errors, reduced quality of care [3], reduced patient satisfaction [4], reduced work effort [5], and leaving medicine entirely [6].

While literature on medicine and healthcare leadership promotes strategies to enhance work meaning and increase physician engagement, data are lacking on the efficacy of these approaches and interventions [7–9]. Furthermore, intervention efficacy may differ among academic faculty depending on their proportional efforts toward patient care, research, and education missions. It is generally unknown how teaching roles may influence work meaning for physicians, but two prior studies found work engagement to be higher for patient care roles than for teaching roles [10, 11]. However, these results were found for traditional academic medical teaching roles such as lecture-based didactics and clinical precepting. But undergraduate medical education is seeing the rise of alternative pedagogical approaches to the traditional lecture that may affect physician work meaning experiences in different ways.

One educational model that is gaining significant traction within medical education is the adoption of learning communities for mentoring and advising, curriculum delivery, community service, and social connections; as of 2012, over half of American Association of Medical Colleges (AAMC) member schools had incorporated learning communities into their medical student educational programs [12–14]. Learning communities are uniquely designed as a relationship-centered educational structure, with groups of students working together longitudinally with one or more faculty mentors. The positive effects of this relationship-centered model on students have already been observed [15]. Learning community models may contribute or detract from faculty work meaning differently from traditional teaching roles. One survey-based study found increased job satisfaction among learning community mentors [16], but specifically how faculty may derive meaning from participating as learning community mentors, and how derived meaning might impact other areas of work has not been described.

We therefore conducted this study to (a) determine the nature of meaning that learning community mentors may experience, (b) understand how learning community mentors-derived meaning may impact other areas of work, and (c) relate our findings to existing literature on work meaning.

## Methods

### Study design

We selected a qualitative phenomenological approach to develop and conduct semi-structured telephone interviews for analysis aimed at better understanding the learning community mentor experience and potential meaning derived from role.

### Protocol development and participant recruitment

From January to June 2018, our research team refined its study question, selected methods, consulted with experts in qualitative study design, and developed the protocol for learning community faculty interviews (Appendix A of the Electronic Supplementary Material). The research interviewers pilot-tested the protocol and recording system during paired interviews of each other. Our team represented four medical schools which all use learning communities for clinical skills instruction. Researchers emailed study details to the cohorts of learning community faculty at their respective schools to solicit volunteers to participate in the telephone interviews; the 25 participants were informed that they would be interviewed by a research colleague from another medical school with curricular learning communities.

### Interviews

A researcher unassociated with the interviewee school conducted each interview (DR, PRW, JMW, MB). We conducted the 25 interviews with two phone calls each: the first for reviewing the consent and answering questions and the second for conducting the interview. No interviews required repeating. We used the RevCall Recorder App and transcription service to record and transcribe the second call (Rev.com, San Francisco, CA). Researchers did not document field notes during the interviews. Participants were not invited to review interview transcripts, but transcripts were reviewed for accuracy by the interviewers. Interviews ranged in length from 7 min and 53 s to 26 min.

### Coding process

We employed the Dedoose software to manage our data (https://www.dedoose.com, Manhattan Beach, CA).

Each research team member independently read the first three interview transcripts and collaboratively created a draft code book by listing possible codes that we consolidated into four main codes. As a group we applied these four codes to an additional interview transcript.

We then distributed the first four interview transcripts plus nine more to the researchers for independent coding. After this round of coding, we discussed potential additional codes and refined our shared mental model of the codes by discussing differences in coding and working towards consensus. We added one more code related to meaning and a sixth code related to negative effects of participation in learning communities for a total of six codes. We re-coded the 13 previously coded interviews using the refined code book.

We then used the refined code book to code the remaining 12 interview transcripts. Additionally, we assigned a second team member to independently code 40% (*n* = 10) of the 25 interview transcripts to ensure coding agreement. During this round of coding we did not identify any new codes; we agreed that there was code saturation and determined that no further interviews were needed. The “I feel connected” and “I am contributing” codes had coding differences among team members. Team members who coded the same interviews resolved their coding differences by telephone discussions. From these discussions, we proposed defining distinctions for the “I am a better professional,” “I am more connected,” and “I am contributing” codes and finalized our code book with definitions. We then re-applied the more clearly understood codes to each of our previously assigned interview transcripts.

### Analysis

We analyzed our qualitative data and determined that each of the six codes represented a distinct theme. We then conducted a relational analysis of our observations in the context of theories and conceptual frameworks published in the literature.

This multi-institution study was deemed exempt by the University of Utah School of Medicine. The University of New Mexico School of Medicine and University of Texas-Southwestern Medical School were required to make an additional exempt determination due to their institution specific IRB requirements. This study was carried out in accordance with the Declaration of Helsinki. There was no potential harm to participants, the anonymity of participants was guaranteed, and informed consent of participants was obtained.

## Results

No study volunteers declined to participate in the interviews after the consent process. The 25 physician study participants from the four medical schools included a mix of women and men, primary and specialty care, and academic rank.

### Codes and themes

We agreed that five of the codes represented distinct themes about meaning faculty derived from learning community participation. The sixth code represented the theme of experiencing harm as a result of serving as a learning community mentor. The six themes are summarized in Tab. 1 of the Electronic Supplementary Material. Representative quotes elaborate each theme.

**I am a better professional. **Faculty described experienced meaning as a sense of having become a better professional in domains such as teaching students and/or residents, advancing or renewing their own clinical skills, and improved relationships with patients.

*I see a true educator [referring to self] now as opposed to just sort of standing in front of a room and lecturing or whatever it may have been before. *(Learning community (LC) Faculty Mentor 15)

*It’s definitely improved my physical exam skills because I am constantly refreshing them by teaching them. *(LC Faculty Mentor 3)

(Referring to before becoming a LC mentor)* “I have really taken to heart about explaining to patients about things in a very simple, straightforward manner so they can understand it which perhaps, I was not quite so aware of [referring to before becoming a LC mentor].” *(LC Faculty Mentor 11).

**I am more connected. **Faculty noted their increased feelings of connection to individual students, the student body, other faculty, the education mission, the institution, and medicine in general.

*The mentoring and learning from students have made me, uh, just intimately much more aware of what students are experiencing. *(LC Faculty Mentor 5)

*I’ve been given a little bit more of a window into the entire experience at the College of Medicine, and have a much better understanding of how the college works. *(LC Faculty Mentor 4)

**I am rejuvenated. **Faculty often expressed that the learning committee experience created a buffer against negative aspects of work, engendered feelings of renewed purpose, or represented a career highlight.

*I find more meaning in coming to work every day and I certainly have a lot more positive experience with my patients as well. *(LC Faculty Mentor 13)

*I tell you it is an absolutely bright spot. Not only of my week but mostly it is an absolute bright spot of my almost in 40 years postgraduate medical career. *(LC Faculty Mentor 2)

Learning community faculty also felt their students reminded them of why they chose a career in medicine and they described renewed hope for the future of medicine.

*It took me back to that time. It kind of makes me, it kind of rekindled all of those feelings. I think that’s a good thing because I think when you forget about all that, I think that’s why physicians burn out. I think that’s when you get jaded in the field. And I think what this does is kind of reminds you, and keeps you young, or brings you back to that time. *(LC Faculty Mentor 7)

**I am contributing. **The “I am contributing” theme related most to feelings that they were improving the future of medicine by contributing to the development of individual students as physicians.

*It’s been both educational and humbling to realize that you’re participating literally in the growth of a clinician, of a physician. *(LC Faculty Mentor 9)

**I am honored. **In this theme faculty experienced validated sense of self when they, as individuals, or through their association with the learning committees, were viewed favorably by students, departments, the promotion process, or other medical schools.

*But kind of like a validation, if you will, of this may not be something that’s as appreciated by my peers more broadly, but it’s appreciated by these young students and aspiring physicians. *(LC Faculty Mentor 23)

*Quite frankly, none of us dreamed that it was going to be this successful … That’s what’s really surprised me, that it has been widely accepted by the students and now emulated across many medical schools. *(LC Faculty Mentor 1)

**I am harmed. **Faculty also mentioned negative aspects of learning community participation such as the time burden of being a learning community mentor and competition with other responsibilities.

*So, it’s a bit of a snub to my partners for me to leave five or 10* *min early to walk to another building because I’m often in charge of the learning community session. *(LC Faculty Mentor 5)

In analyzing the transcript text surrounding coded comments related to experienced harm, we often found that learning committee faculty who mentioned a negative aspect of learning committees did so in the context of describing how benefits of participation outweighed the negative aspects.

### Relational analysis

**Fig. 1:**
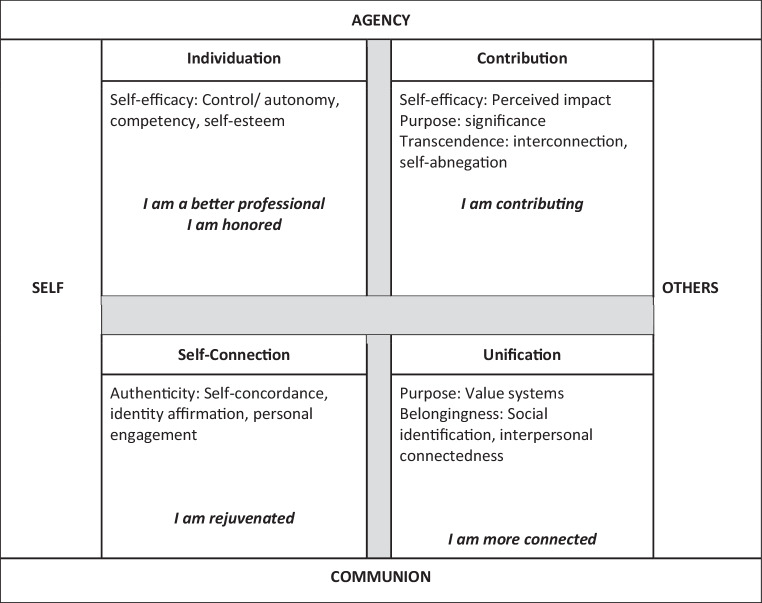
Categorization of learning community faculty meaning themes by Rosso’s theoretical framework for pathways to meaningful work [17]

After analyzing our themes, we explored them in the context of Rosso et al.’s theoretical framework on the pathways to meaningful work [17]. In this theoretical framework developed from review and integration of literature on sources of meaning of work and the mechanisms by which work becomes meaningful, Rosso et al. described two perpendicular spectral axes of agency-communion and self-others defining four major pathways to meaning: Individuation (self-agency), Contribution (other-agency), Self-Connection (self-communion), and Unification (other-communion). The authors emphasized that these pathways are not mutually exclusive and they hypothesized that work that simultaneously activates more than one pathway could contribute to stronger perceptions of meaningfulness. As depicted in Fig. 1, we found that we could map our themes of derived work meaning to Rosso’s theoretical model.

For our “I am a better professional” and “I am honored” themes, we reviewed interview passages in detail to ultimately place these two codes into the Individuation (self-agency) quadrant for the reasons that comments from these themes expressed a drive to differentiate oneself in positive or negative terms or reflected the meaningfulness of actions that define and distinguish the self as valuable and worthy (e.g. “I am honored” *I was pleasantly surprised by being invited to be a mentor *[LC Faculty Mentor 17]) [17]. Our “I am more connected” theme fit well into the Unification (other-communion) quadrant, which expresses the meaningfulness of actions that bring individuals into harmony with other beings or principles [17]. Our “I am contributing” theme fit directly into the Contribution (other-agency), where the focus is on a drive to assert oneself in the service of other individuals, groups, collectives, organizations, or higher powers [17]. We found that the “I am rejuvenated” theme fit most closely with the Self-Connection (self-communion) quadrant where meaningfulness is assigned to actions that bring individuals closer into alignment with the way they see themselves (e.g. *“reinforces … who are we”* [LC Faculty Mentor 2], *“reminding myself how much I love what I do, and the things that I love about what I get to do, and the privileges of being a physician”* [LC Faculty Mentor 4]). As threats to experienced meaning, comments from the “I am harmed” theme mapped across the four quadrants of the theoretical framework.

## Discussion

Our study contributes to the growing body of literature on work meaning generally and on work meaning more specifically among physicians in academic medicine. Our findings also deepen understanding of potential benefits and harms experienced by learning community faculty in longitudinal teaching relationships with students.

### Relationship to existing literature

A particular strength of the Rosso et al. theoretical framework for pathways to meaningful work is that it was synthesized from broad sources of psychology of work literature aimed at understanding work meaning, work motivation, job engagement, and burnout. Because this model is so comprehensive, it is not surprising that our themes of meaning experienced by learning community faculty are readily mapped to it. But general models may be less able to provide practical contextualizations of observations. We therefore also sought other conceptual theories as potential lenses through which we might consider our findings, and we found that our themes related well to self-determination theory (SDT) [18, 19]. SDT attributes motivation for human behavior to that beyond physical needs and asserts that humans have a need for autonomy, competence, and relatedness. These SDT needs aligned well with the Rosso et al. pathways model and, by extension, our themes. Needs for autonomy and competence map to Rosso’s Individuation (self-agency) quadrant and our “I am a better professional theme.” The “I am honored” theme may relate to the SDT observation that social-contextual events that provide feedback suggesting competence may enhance intrinsic motivation provided that there is a sense of autonomy [18]. The SDT need for relatedness maps to Rosso’s Contribution (other-agency) quadrant and our “I am contributing” theme as well as Rosso’s Unification (other-communion) quadrant and our “I am more connected” theme. Importantly the “I am harmed” theme that arose in our analysis represents a potentially significant threat to multiple pathways to meaningful work in Rosso and SDT models. In particular, our “I am contributing” and “I am more connected” themes are important corroborations of both SDT and Rosso et al.’s works which assert that, beyond self-oriented motivation, externally-oriented processes (i.e. relatedness, unification, and contribution) are also important contributors to perceived work meaningfulness [17–19].

In potential contrast to two prior quantitative studies of academic faculty teaching roles which found lower engagement in teaching roles compared with clinical roles [10, 11], we identified five themes of meaning faculty derive through their work as learning community mentors. Acknowledging that direct comparison cannot be made between our two studies due to methodologic differences, Van den Berg et al. found that combining teaching and patient care tasks was a distraction to engagement in teaching [10]. By contrast our faculty described how engagement with education through learning communities contributed synergistically to their clinical work and the meaning they derived from it. Again, acknowledging methodologic differences, whereas Scheepers et al. found that work as a clinician was not positively correlated with teaching performance, faculty in our study commented, conversely, that they felt they were better clinicians because of their learning community educational work [11]. The centrality of longitudinal relationships to the learning community model may be an important factor to consider in future work to understand potential interactions between the multiple roles of academic physicians and the impact on experienced work meaning.

The survey-based study by Wagner et al. found a 96% rate of increased satisfaction and happiness with their jobs and 87% rate of improved sense of belonging to their institutions as a result of learning community participation [16]. Response patterns suggested two primary factors contributing to faculty satisfaction: Campus Engagement and Skills Development. Our methodologically different qualitative study provides corroboration of these findings with our themes of “I am a better professional” and “I am more connected” directly paralleling their two factors; however, our qualitative approach allowed the emergence of additional themes that may not have been considered in the Wagner et al. survey development process. Our qualitative approach also allowed us to explore detrimental effects of learning community participation that could be explored in further quantitative research about learning community faculty experiences.

### Limitations

Our study has a number of limitations. First, though we primarily approached our coding process in the conventional manner of deriving and defining our codes from the collected data, prior to finalizing our interview questions, we conducted a literature review on work meaning and prospectively identified Rosso et al.’s theoretical integration and review of work meaning as a potential theoretical framework [17]; this prior consideration of a theoretical framework has some features of directed content analysis [20]. We attempted to mitigate bias related to this prior consideration by avoiding designing our interview around this framework and by approaching our data collection inductively with the intent of determining retrospectively if it fit Rosso’s theoretical framework. It is possible that our codes were influenced by this prior consideration; this is reflected in the broad nature of our codes and their direct mapping to our themes. However, the titles for our codes included words or similes used frequently by interviewees (e.g. contributing, connected, rejuvenated, honored, better).

Another limitation of the study is that participants were volunteers rather than randomly selected subjects. Participants may have been more engaged in learning community work than those who did not volunteer for the study. Additionally, we collected our data through interviews that we conducted ourselves; this could introduce an element of bias. We tried to mitigate this potential bias by standardizing our interview protocol, conducting multiple iterations of code consensus building, and not interviewing faculty from our home institutions.

Finally, all of our participating schools employed curricular learning communities; thus, our findings may not be applicable to learning community models designed for other purposes. Future work could explore how aspects of faculty work meaning vary between different learning community models.

### Significance of findings

Our findings are important in suggesting that learning community participation offers a number of pathways to meaningful work. The congruence of our themes of learning community faculty experience with both an integrated work meaning theoretical framework and a framework relevant to work motivation literature are important to evolving understanding about pathways to enhanced work meaning for physicians.

Our results may suggest further areas requiring investigation related to the learning community model and its impact on experienced physician work meaning. Deliberate interventions to capitalize on learning community model contributions to faculty work meaning might explore how formalizing student and institutional learning community faculty recognition enhance the learning community faculty meaning through the “I am honored” theme; though SDT suggests that external attempts to motivate behavior can result in response ranging from amotivation to active commitment [18]. Understanding potential harms experienced by learning community faculty may suggest targeted areas for improving learning community implementation such as mitigating competition with clinical responsibilities or preventing interference with priority personal time away from work.

As learning communities become an increasingly common among medical schools, it is important to continue to expand our understanding of this educational model. We have already seen the learning community literature expanding beyond early descriptive and demographic studies about learning communities toward general outcomes for learners and faculty [12–16]. Our work is a first step a deeply exploring the learning community educational model within medical schools.

## Caption Electronic Supplementary Material

Appendices

Table 1 Final themes and sub-themes arising from codes

## References

[1] Maslach C, Schaufeli WB, Leiter MP (2001). Job burnout. Annu Rev Psychol.

[2] Kahn WA (1990). Psychological conditions of personal engagement and disengagement at work. Acad Manag J.

[3] Williams ES, Manwell LB, Konrad TR, Linzer M (2007). The relationship of organizational culture, stress, satisfaction, and burnout with physician-reported error and suboptimal patient care: results from the MEMO study. Health Care Manag Rev.

[4] Haas JS, Cook EF, Puopolo AL, Burstin HR, Cleary PD, Brennan TA (2000). Is the professional satisfaction of general internists associated with patient satisfaction?. J Gen Intern Med.

[5] Shanafelt TD, Mungo M, Schmitgen J (2016). Longitudinal study evaluating the association between physician burnout and changes in professional work effort. Mayo Clin Proc.

[6] Landon BE, Reschovsky JD, Pham HH, Blumenthal D (2006). Leaving medicine: the consequences of physician dissatisfaction. Med Care.

[7] Shanafelt TD (2009). Enhancing meaning in work: a prescription for preventing physician burnout and promoting patient-centered care. JAMA.

[8] Shanafelt TD, Noseworthy JH (2017). Executive leadership and physician well-being: nine organizational strategies to promote engagement and reduce burnout. Mayo Clin Proc.

[9] West CP, Dyrbye LN, Erwin PJ, Shanafelt TD (2016). Interventions to prevent and reduce physician burnout: a systematic review and meta-analysis. Lancet.

[10] van den Berg BAM, Bakker AB, ten Cate TJ (2013). Key factors in work engagement and job motivation of teaching faculty at a university medical centre. Perspect Med Educ.

[11] Scheepers RA, Arah OA, Heineman MJ, Lombarts KMJMH (2015). In the eyes of residents good supervisors need to be more than engaged physicians: the relevance of teacher work engagement in residency training. Adv Health Sci Educ Theory Pract.

[12] Smith S, Shochet R, Keeley M, Fleming A, Moynahan K (2014). The growth of learning communities in undergraduate medical education. Acad Med.

[13] Ferguson KJ, Wolter EM, Yarbrough DB, Carline JD, Krupat E (2009). Defining and describing medical learning communities: results of a national survey. Acad Med.

[14] Osterberg LG, Goldstein E, Hatem DS, Moynahan K, Shochet R (2016). Back to the future: what learning communities offer to medical education. J Med Educ Curric Dev.

[15] Smith SD, Dunham L, Dekhtyar M (2016). Medical student perceptions of the learning environment: learning communities are associated with a more positive learning environment in a multi-institutional medical school study. Acad Med.

[16] Wagner JM, Fleming AE, Moynahan KF, Keeley MG, Bernstein IH, Shochet RB (2015). Benefits to faculty involved in medical school learning communities. Med Teach.

[17] Rosso BD, Dekas KH, Wrzesniewski A (2010). On the meaning of work: a theoretical integration and review. Res Organ Behav.

[18] Ryan RM, Deci EL (2000). Self-determination theory and the facilitation of intrinsic motivation, social development, and well-being. Am Psychol.

[19] ten Cate OTJ, Kusurkar RA, Williams GC (2012). How self-determination theory can assist our understanding of the teaching and learning processes in medical education. AMEE Guide No. 59. Med Teach.

[20] Hsieh HF, Shannon SE (2005). Three approaches to qualitative content analysis. Qual Health Res.

