# Supplementary material for: The learning community faculty experience: how longitudinal relationships with learners enhance work meaning
Source: Perspect Med Educ. 2020 Aug 20;9(6):343–9. doi: 10.1007/s40037-020-00614-z (PMC7718352; doi:10.1007/s40037-020-00614-z)
Supplement: Supplementary file 1 — Appendices [file 40037_2020_614_MOESM1_ESM.docx]

**Table 1: Final Themes and Sub-Themes Arising from Codes**

| **Themes** | **Sub-Themes** |  |
| --- | --- | --- |
| I am a better professional | I am better at:   - Teaching - Mentoring - Being a doctor   - Performing the physical examination   - Communicating with patients and colleagues. | |
| I am more connected | I have deeper relationships with:   - The school of medicine - Colleagues in medicine - Colleagues from interprofessional disciplines - Students (professional and personal)   I have a better understanding of students’:   - Parents - Family - Growth - Medical school experience   I have a better understanding of my colleagues | |
| I am rejuvenated | I am staying young/feeling young again  I have a renewed sense of purpose  I now have hope for future of medicine  I have a broader perspective on medicine  I feel invigorated in research  I feel fulfilled  I have increased well-being | |
| I am contributing | I am contributing to:   - Individual student development by:   - Teaching   - Advising   - Mentoring   - Counseling - A legacy - A better future of medicine | |
| I am honored | My ego is validated through:   - Recognition from LCs/Department/University - Gratitude from students | |
| I am harmed | I have experienced:   - Reduced productivity - Increased work at home - Time away from family - Increased burnout | |
