# Supplementary material for: The learning community faculty experience: how longitudinal relationships with learners enhance work meaning
Source: Perspect Med Educ. 2020 Aug 20;9(6):343–9. doi: 10.1007/s40037-020-00614-z (PMC7718352; doi:10.1007/s40037-020-00614-z)
Supplement: Supplementary file 2 — Table 1 Final themes and sub-themes arising from codes [file 40037_2020_614_MOESM2_ESM.docx]

**Appendices**

**Appendix A: Interview Instructions and Question Protocol**

Phone Call 1:

“I would like to ask you a few questions to understand how medical student learning communities gives you meaning. I will record this interview, but please know that once the audio is transcribed to text the recording will be deleted. However, there could be a potential loss of confidentiality if there was a breach of security.

Your name will not be linked to the transcription. A team of trained qualitative researchers will be the only people with access to the transcription. Participating in this study will have no impact on continuing as a learning community core faculty member in the course and the course directors will not have access to a list of participants.

Do you have any questions from the consent document or about this interview process before we begin?

I will hang up now and will call you back while recording. Are you ready?”

HANG UP

Phone Call 2:

CALL INTERVIEWEE BACK WITH REV.COM APP

“This is WWW INTERVIEWER interviewing a faculty member who is a learning community faculty/mentor at ZZZ INSTITUTION.”

1. “My first question for you is, what, if any, changes have you experienced in your overall work as a faculty member? That is, how has your work as a learning community faculty member impacted your professional life both positively and negatively?”

Follow-up with clarifying questions:

- “Can you tell me more about what you mean when you say AAA?”
- “Do you have any specific examples or anecdotes about BBB?”
- “You mentioned CCC; how is that different compared to before you were a learning community faculty member?”

2. “You identified some changes in your overall work; what, if any, changes have you experienced in the way you approach your work as a result of your participation as a learning community faculty (including non-learning community days/times)?”

Follow-up with clarifying questions:

- “Can you tell me more about what you mean when you say AAA?”
- “Do you have any specific examples or anecdotes about BBB?”
- “You mentioned CCC; how is that different compared to before you were a learning community faculty member?”

3. “Finally, what, if any, changes have you experienced in your views/attitudes about your work as a result of your participation as a learning community faculty? Specifically, have there been any changes to what motivates and inspires you?”

Follow-up with clarifying questions:

- “What have you been surprised by?”
- “Can you tell me more about what you mean when you say AAA?”
- “Do you have any specific examples or anecdotes about BBB?”
- “You mentioned CCC; how is that different compared to before you were a learning community faculty member?”

4. “Are there any other changes you haven’t shared related to your participation as a learning community faculty? That is, is there anything else you want to share today?”

“Thank you very much for allowing me to interview you; I enjoyed hearing about your experience as a learning community faculty!”
